# Complexities in the Diagnosis and Management of Anti-Hu Antibody-Associated Paraneoplastic Syndrome

**DOI:** 10.7759/cureus.69457

**Published:** 2024-09-15

**Authors:** Yovan Ram Kurrun Sumaruth, Elmahi Mohammed, Emma Louise Robinson, Manasi Limaye

**Affiliations:** 1 Internal Medicine, North Manchester General Hospital, Manchester, GBR

**Keywords:** small-cell lung carcinoma, mesenteric mass, presacral mass, intravenous immunoglobulins (ivig), paraneoplastic neurologic syndrome, anti-hu

## Abstract

Anti-Hu is the most commonly associated antibody in paraneoplastic syndromes (PNS) - mainly secondary to small cell lung cancer (SCLC), breast cancer, thymoma, and lymphoma. This case is about a 65-year-old female patient presenting with slurred speech, headache, and loss of balance for one day. On examination, she was found to have downbeat and bilateral gaze-evoked nystagmus, dysarthria, and bilateral intention tremors. The rest of the neurological examination was unremarkable. Upon investigation, a CT scan showed a pre-sacral mass and a PET scan showed a lobulated soft tissue mesenteric mass at L5/S1, thought to possibly be a gastrointestinal stromal tumour, and mediastinal lymph nodes including right lower pre-tracheal, subcarinal and right hilar lymph nodes. Additionally, paraneoplastic antibody testing was positive for anti-Hu antibodies. She was given a five-day course of intravenous immunoglobulin without significant clinical improvement. The patient was discharged on a fast-track pathway and did not undergo chemotherapy, radiotherapy or surgical resection as the primary tumour could not be diagnosed.

Paraneoplastic antibodies are a family of autoantibodies occurring as a result of malignancy that act to recognize antigens in the brain, resulting in a variety of neurological manifestations. Despite well-known literature on this entity, PNS is notoriously difficult to diagnose and manage. The first step in the management of PNS is to treat the underlying malignancy. Beyond this, the other key component of PNS treatment is immune modulation which may involve immunosuppression with high-dose corticosteroids, IV immunoglobulins, plasma exchange or plasmapheresis. It is therefore important for PNS to be diagnosed early and to adopt a comprehensive multidisciplinary approach to improve the outcomes of those presenting with PNS.

## Introduction

Paraneoplastic syndromes (PNS) are disorders which are associated with cancer. They do not, however, have a direct effect on the tumour mass or its metastases, rather the malignant cells produce these autoantibodies which in turn affect multiple organ systems [1]. They more commonly occur with malignancies such as small cell lung cancer (SCLC), breast cancer, thymoma and lymphoma. PNS presents with diverse symptoms which usually precede the manifestation of the associated cancers, thus leading to difficulty in the early diagnosis of PNS and a delay in intervention in the early stages of malignancy [2].

The incidence of paraneoplastic neurologic syndromes (PNS) is rare and affects less than 1% of cancer patients overall, with varied incidence depending on the type of primary tumour and manifestation of PNS [3]. Paraneoplastic antibodies are a family of autoantibodies that occur as a result of malignancy and act to recognise antigens in the brain, resulting in a variety of neurological manifestations. This mechanism functions via cell-mediated immunity as T cells attack not only the tumour cell antigens but also antigens in normal cells that are similar in structure [4].

Anti-Hu antibodies (also known as anti-neuronal nuclear antibody-type 1 (ANNA-1)) were the first recognised autoantibody markers of small-cell lung cancer [5]. Several other groups of autoantibodies have also been recognised, including ANNA-2 (Ri), Purkinje cell cytoplasmic antibodies (PCCA or Yo), and anti-GAD. The neurological manifestations of these paraneoplastic autoantibodies may differ based on the mechanism of antigen recognition. For example, anti-Hu antibodies are reactive with all neuronal nuclei, particularly Purkinje cells of the cerebellar cortex. Hence anti-Hu antibodies lead to a clinical manifestation of subacute cerebellar degeneration due to this cross-reactivity with cerebellar antigens [4]. Anti-Hu is among the most well-characterised of the paraneoplastic autoantibodies and is the type most frequently seen in patients with PNS. About 2% of cancer patients are positive for anti-Hu and, of these patients, most have SCLC as the primary tumour. Furthermore, the detection of anti-Hu antibodies is a predictor of a poorer prognosis for these patients [6].

Testing for paraneoplastic autoantibodies can be carried out using tissue-based assays and confirmatory results produced by immunoblotting or cell-based assays [7]. Detection of these antigens serves as a potential diagnostic tool but must always be considered in an integrated context with the clinical manifestation of the syndrome. However, despite extensive literature on this entity, correctly identifying and managing the neurological manifestations associated with anti-Hu antibodies remains challenging [6].

## Case presentation

**Figure 1:**
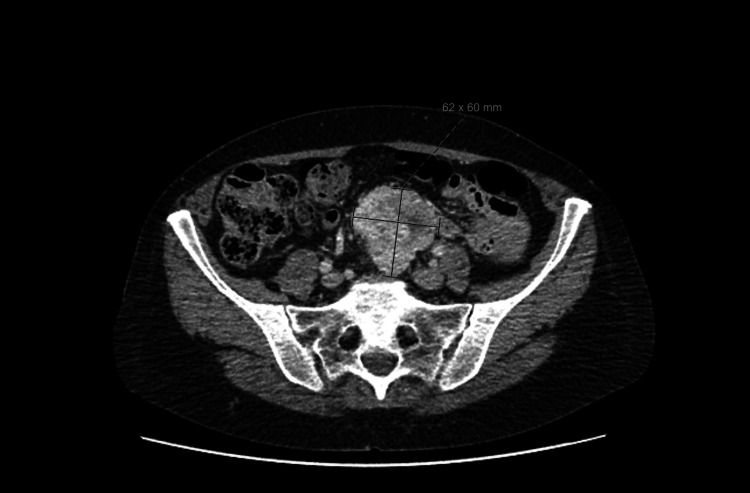
CT Scan showing 6 x 6 x 5.5cm presacral mass which appears to be small bowel in origin

A 65-year-old female presented with a five-week history of intermittent nausea and vomiting as well as a one-week history of constipation. CT abdomen and pelvis revealed a 6x6x5.5cm presacral mass whose appearance was in keeping with a carcinoid tumour (Figure 1).

**Figure 2:**
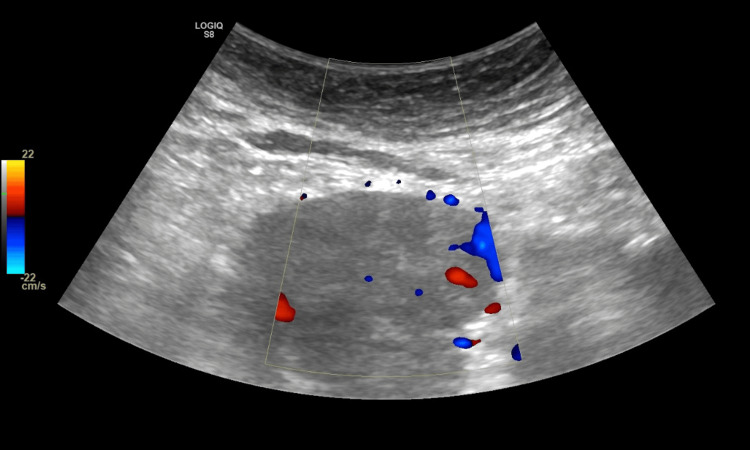
Abdominal ultrasound showing mass. Several mesenteric vessels can be identified anterior to the lower abdominal mass.

The patient was referred to both the general surgery and acute oncology teams and was then discharged with a plan for an outpatient MRI pancreas and CT thorax scan. After one week, the patient presented again to the emergency department with complaints of slurred speech, headache and loss of balance for one day. On examination, she was found to have both downbeat and bilateral gaze-evoked nystagmus, dysarthria and bilateral intention tremors. The rest of the neurological examination was unremarkable. CT head did not reveal any acute abnormalities. It was decided to perform an ultrasound-guided biopsy of the abdominal mass previously found on CT (Figure 2).

**Figure 3:**
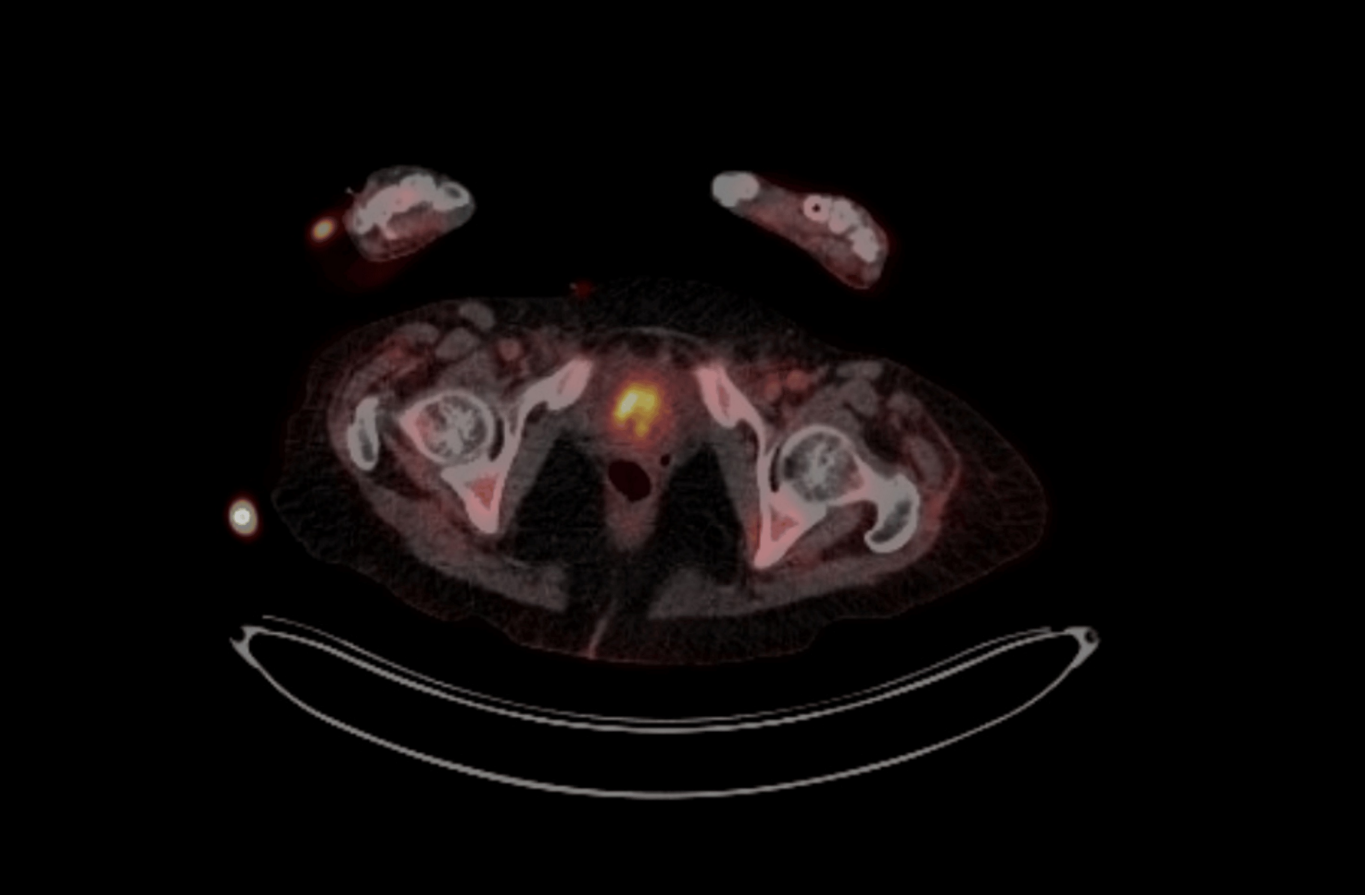
PET scan showing a lobulated soft tissue mesenteric mass at the level of L5/S1, demonstrating mixed metabolic activity with areas of high and low fluorodeoxyglucose uptake

This was unsuccessful due to several mesenteric vessels anterior to the mass. The only window to approach this mass anteriorly was via the left side, however, the superior epigastric vessel was also within the site of approach, therefore the procedure was not performed. A neurology referral was completed and it was advised to treat with IV Pabrinex as the symptoms resembled Wernicke’s encephalopathy. This treatment with Pabrinex did not lead to any clinical improvement. A lumbar puncture was performed on the advice of the neurology team given there had been no response to any treatments trialled to date. PET scan showed a lobulated soft tissue mesenteric mass at L5/S1 (Figure 3), thought to possibly be a gastrointestinal stromal tumour, and mediastinal lymph nodes including right lower pre-tracheal, subcarinal and right hilar.

Additionally, the paraneoplastic antibodies test was positive for anti-Hu antibodies. In light of these results, the multidisciplinary team (MDT) decided to treat the patient with a five-day course of intravenous immunoglobulin (2 g/kg divided over the five days, i.e. 400mg/kg once daily for five days).

However, there was no significant clinical improvement; the patient was still nystagmic and remained ataxic. The patient remained too unwell to proceed with an endobronchial ultrasound-guided biopsy. The patient was discharged on a fast-track pathway and could not undergo chemotherapy, radiotherapy or surgical resection. 

## Discussion

As previously highlighted, PRN is notoriously difficult to diagnose and manage, mostly due to the complex immunologic and non-immunologic mechanisms at play and also due to the rarity of these syndromes, meaning the majority of clinicians do not have direct clinical experience with such presentations. They are also essentially a diagnosis of exclusion in the first instance and hence require more extensive workup before diagnoses can be made [4]. In this case, the patient first presented with intermittent nausea and vomiting which, in the absence of an already diagnosed malignancy, did not raise suspicion of a PNS as an immediate differential diagnosis. Initial workup for this patient revealed a 6x6x5.5 cm possible carcinoid tumour on CT and further outpatient investigations were arranged. The patient then presented again to the emergency department with a clinical picture of subacute cerebellar degeneration which, as highlighted, can closely mimic the presentation of Wernicke’s encephalopathy. The procedural complexities of performing an ultrasound-guided biopsy of the mass seen on CT meant that a definitive diagnosis of the potential primary malignancy was not possible at this stage. This is important as the first step in the management of paraneoplastic syndromes is to treat the underlying malignancy. Beyond this, the other key component of PNS treatment is immune modulation which may involve immunosuppression with high-dose corticosteroids, IV immunoglobulins, plasma exchange or plasmapheresis [4]. Early intervention with immunomodulatory therapies is associated with better outcomes [8]. Active treatment of the underlying tumour in conjunction with immunotherapy is also thought to contribute to positive therapeutic outcomes [9]. It is worth noting, however, that there are a limited number of studies available that evaluate immunotherapy alone versus treatment of the tumour alongside immunotherapy. This mostly seems to be due to limited cohorts of patients with PNS in whom a tumour is not yet identified or in whom anti-tumour therapy is not an option; it represents an important area for further study. 

Further investigations for this patient revealed positive anti-Hu antibodies, lumbar puncture revealed CSF with elevated protein and cell counts and a PET scan showed a possible gastrointestinal stromal tumour with significant mediastinal lymph node involvement. From the outcomes of multiple MDT discussions involving surgical, neurology, acute oncology and internal medical teams the patient was given courses of both high-dose dexamethasone and IV immunoglobulin. Despite some possible subjective improvement of symptoms with dexamethasone, namely improved oral intake, speech and decreased lethargy, there was no significant clinical response to either dexamethasone or IV immunoglobulin. Given the patient’s rapid deterioration to the current clinical condition, endobronchial ultrasound was considered not to be in the patient’s best interests, pending re-evaluation should there be a significant clinical improvement in response to treatment with IV immunoglobulin. The role of IV immunoglobulins in the treatment of PNS is mostly to stabilise patients rather than provide a noticeable clinical improvement [8]. In the majority of cases, immunotherapy alone is unlikely to be effective [9]. It is likely in this case that the degree of neurological degeneration prior to treatment indicated that the window for stabilisation with IV immunoglobulins had already passed. The patient, unfortunately, also had deteriorated to such an extent that they were unable to undergo further investigation of the primary tumour or to be a candidate for active treatment of the malignancy, thus it was decided via MDT discussions and discussions with the patient and their family that a palliative approach was the best approach at this stage. 

In terms of classification of PNS, this patient would have been classified as having definite PNS due to the presence of a classical syndrome and the presence of a tumour; however, it is worth noting that, even in the absence of a tumour, this patient had well-characterised onconeuronal antibodies in the presence of a classical syndrome so would have been categorised as having definite PNS at this stage either way [10]. It is worth noting that the classification of PNS into definite and possible is likely not to have a direct impact on clinical outcomes, aside from potentially aiding in earlier diagnosis, but rather aids in a more uniform reporting of these cases which may help to focus research in such a rapidly evolving field. 

## Conclusions

This case highlights the complexities in diagnosing and managing paraneoplastic syndromes, particularly those involving anti-Hu antibodies. The patient presented with non-specific symptoms that evolved into severe neurological deficits, complicating early diagnosis. The presence of a pre-sacral mass and positive anti-Hu antibodies confirmed PNS but the patient’s frailty limited therapeutic options. Key learning points include the necessity for a low threshold of clinical suspicion, the importance of early and comprehensive diagnostics and the critical role of a multidisciplinary approach. Early intervention with a combined approach of treatment of the primary tumour and immunotherapy is vital for better outcomes. This case emphasises the need for further research on optimal management strategies and highlights the challenges in treating PNS in advanced stages.
